# Spanish youth is emigrating: A bibliometric approach to the media coverage

**DOI:** 10.1371/journal.pone.0198423

**Published:** 2018-06-20

**Authors:** Clara Selva, Aniol Recordà

**Affiliations:** 1 Department of Social Psychology, Universitat Autònoma de Barcelona, Bellaterra, Catalunya, Spain; 2 Department of Social Psychology, Independent Researcher, Sabadell, Catalunya, Spain; University of Toronto, CANADA

## Abstract

Recent years have witnessed the emigration of young Spanish people searching for labor opportunities. A decade after the beginning of the worldwide economic crisis in 2007, the rapid deterioration of living conditions and lack of opportunities for personal development combined with the breakdown of professional expectations have led thousands of young people to emigrate from Spain, creating the so-called youth exodus. The press has paid recurrent attention to this phenomenon, often using eye-catching headlines such as ‘Brain Drain’. Given the regular interest of the media in this phenomenon, the objective of this research is to analyze the media coverage of the drain of Spanish talent capital, or the emigration of young Spanish people seeking a better future, to create a distributive map that defines the characteristics and trends of this coverage. The analyzed corpus comprises 346 articles from eight Spanish and eleven international newspapers. The articles were coded based on descriptive variables (i.e., *author*, *publication year*, *newspaper* and *language*) and categorical variables (i.e., *section*, *method*, *approach to the phenomenon*, *assessment of the phenomenon* and *overview of the phenomenon*). The results indicate a significant increase in press coverage over the past few years and reveal associations between *assessment of the phenomenon* and *year* and between *assessment of the phenomenon* and *section*. As a result of this research, new investigative lines are unveiled regarding the social construction of the phenomenon in the media and the identity and individual construction of the ‘truncated careers’ of young Spanish people.

## Introduction

Destination, Europe. A greater chance of finding a job, the ease of utilizing obtained degrees, the proximity, and the free movement of workers have made certain European countries a destination for thousands of young people (and some not-so-young people) who have decided to pack their bags and become the protagonists of the most important Spanish emigration wave in recent times [1]. A decade after the beginning of the worldwide event known as the great recession [2], unquestionably poor economic conditions and their broad impact (beyond the scope of significance of the recession itself) led to the simultaneity and coexistence of economic, social, cultural, political and values crises, among others [3].

Although the crisis has been felt all over Europe, different intensities of the problem in each country have created diverse social constructions of the recession [4]: in countries that have suffered a lesser economic impact, speeches have focused more on economic and financial aspects, whereas in countries that suffered a higher economic impact, a more diversified discourse has taken place, focusing not only on economic issues but also on social, labor, political and/or cultural issues. The latter group includes Spain, Greece and Italy; Spain is particularly notable for its highly suspicious attitude towards the government and public institutions and for the existence of an alarming new dimension: the political legitimacy crisis [4].

One of the most noticeable consequences of the crisis in Spain is the dramatic deterioration of living conditions [5, 6] with reiterated falls of the gross domestic product (GDP) between 2009 and 2013 (including significant decreases of 3.6% in 2009 and 2.9% in 2012), a tripling of the unemployment rate (rising from 7.93% in the second quarter of 2007 to 26.94% in the first quarter of 2013 situated in the second quarter of 2017 at 17.22%), and a sharp increase in the poverty risk and social exclusion rate (from 23.8% in 2008 to 27.3% in 2013, situated in 2017 at 24.7%).

The crisis has affected the entire society, but the effects have been particularly harsh among young people, a group characterized by endemic conditions of economic precariousness and labor instability [7, 8]. The consequences are tangible: data from the Ministerio de Empleo y Seguridad Social (Ministry of Social Welfare and Employment) [9] show that from 2007 to 2011, 2,008,577 Spanish workplaces staffed by people under 30 years old shut down, eliminating 36.6% of the existing jobs. Within the same period, the youth unemployment rate for people between 16 and 24 years old reached 55.5% (in comparison, the average European rate in the same time frame was 23.6%), being 44.4% at the end of 2016 [6, 10]. However, unemployment is not the only problem faced by young people [11]; rather, the labor reality for this group continues to be characterized by temporary, part-time or under-skilled employment. The increased precariousness of the labor market and the real rate of underemployment—factors that are far too complex to be detected by statistical data—contribute to the expanding salary gap between younger and older workers [12]. On a psychological level, when temporality is attached to high unemployment rates, as in the case of Spain, unintentional temporary employment becomes more like unemployment than an actual occupation because it involves high levels of insecurity, uncertainty and hopelessness, which increase the risks of marginality and social exclusion among young people. Therefore, unintentional temporary employment causes an even sharper reduction of occupational well-being than does unemployment [13].

The high level of precariousness and the demand for flexibility in young people’s work and social lives have led to delays in certain transitional thresholds to adult life (such as leaving the parental home or having children), which is one strategy for addressing these difficulties. This phenomenon, known as ‘youth prolongation’ [14], implies not only a delay in the age at which the transition to adult life is completed but also a shift in the narratives [15] of young people and in the identity construction of the social category ‘being young’ in order to align them with contemporary trends [16]. In this new reality, linear trajectory models [17] in which professionalization or academic education ensures the achievement of a job have become outdated [18], giving way to mismatches between labor market demands and the education of young people (i.e., ‘over-qualification’) or incompatibilities between executed tasks and academic education.

In this context, the phenomenon of ‘truncated careers’ emerges [19]. The term ‘truncated careers’ refers to the conflict between the expectations encouraged by (or in) young people throughout their academic educational journeys and the later (negative) reality they experience when they enter the labor market; as a result of this conflict, the trajectory, or professional career, of young people might be truncated by the volatile and unsteady situation caused by the recession of previous years. Different action strategies, such as migration or resilience, may be derived as ways to address the situation of ‘truncated careers’. In sum, the waste of talent that generated this phenomenon has its roots in young people, but its effects go further, reaching the economic, social and educational spheres of the country.

Associated with ‘truncated careers’ is a new dimension of precariousness: the broad emigration of qualified young people also known as ‘brain drain’ [12]. This drain has at least two mid-term consequences: an absence of labor force replacements and the aging of the population, which will directly affect the public pension system in the future [20]. Meanwhile, the lack of decent job opportunities that enable a fulfilled and autonomous life in Spain has driven thousands of young people to emigrate between 2012 and 2016, leading to a loss of approximately 1,600,000 Spanish residents under the age of 34 years [21, 22].

Despite the lack of accurate and reliable data, which prevents an exact measurement of the extent of the phenomenon (many young people do not formally communicate their departure; consequently, the number of young emigrants may be greater), it is clear that Spain is experiencing a ‘youth exodus’ [21]. The short- or mid-term absence of job opportunities that allow personal development combined with the breakdown of professional expectations has induced the young (highly trained) population to emigrate to areas outside the Spanish borders, where they often work in jobs that are completely unrelated to their education. The data (see Table 1) confirm that the youth exodus has intensified and solidified as a migration phenomenon since the beginning of the crisis, with 19,889 more emigrations registered in 2016 than in 2007 [10]. In this emigration wave, women predominate over men, especially in the 20- to 30-year-old group. This difference can be explained by the generalization and feminization of university and post-university education and by the increased demand for workers in specific niche markets in the destination countries that are predominantly feminine (e.g., personal and sanitary services in the United Kingdom and Germany) [23].

**Table 1:** Spanish emigration by year, gender and age group.

	From	15 to 19	20 to 24	25 to 29	30 to 34	
2016	Men	2,016	2,417	4,806	6,072	
	Women	1,968	2,900	5,780	6,181	
	Total	3,984	5,317	10,586	12,253	32,140
2015	Men	1,967	2,630	5,810	7,127	
	Women	2,089	2,997	6,482	7,136	
	Total	4,056	5,627	12,292	14,263	36,238
2014	Men	1,718	2,207	4,890	6,031	
	Women	1,812	2,678	5,327	6,116	
	Total	3,530	4,885	10,217	12,147	30,779
2013	Men	1,604	1,943	4,134	5,449	
	Women	1,500	2,254	4,429	5,274	
	Total	3,104	4,197	8,563	10,723	26,587
2012	Men	1,185	1,510	3,440	4,394	
	Women	1,231	1,742	3,527	4,181	
	Total	2,416	3,252	6,967	8,575	21,210
2011	Men	1,067	1,367	3,330	4,334	
	Women	1,065	1,563	3,498	4,406	
	Total	2,132	2,930	6,828	8,740	20,630
2010	Men	772	1,006	2,413	3,072	
	Women	758	1,177	2,485	3,058	
	Total	1,530	2,183	4,898	6,130	14,741
2009	Men	752	858	2,139	2,697	
	Women	712	963	2,316	2,882	
	Total	1,464	1,821	4,455	5,579	13,319
2008	Men	717	906	2,273	2,525	
	Women	686	1,003	2,544	2,671	
	Total	1,403	1,909	4,817	5,196	13,325
2007	Men	486	815	2,220	2,274	
	Women	470	1,052	2,478	2,456	
	Total	956	1,867	4,698	4,730	12,251
2006	Men	389	717	1,754	1,748	
	Women	397	911	2,192	2,070	
	Total	786	1,628	3,946	3,818	10,178
2005	Men	357	699	1,772	1,475	
	Women	369	956	2,200	1,902	
	Total	726	1,655	3,972	3,377	9,730

Data extracted from Eurostat [10]

Europe is the most common destination for young Spanish emigrants, and the United Kingdom, Germany and France are the largest recipients of this talent capital [10], with the UK in the leading position. Data collected by the municipal population census, which registers only disclosed emigrations, indicate that 9,531 young people from 15 to 34 years old emigrated to one of these three countries in 2016 (5,604 more people than in 2008) shortly after the beginning of the crisis (see Table 2). However, it is estimated that approximately 68.4% of young Spanish emigrants do not disclose their departures to the Spanish consulate [3]; thus, Spain might be experiencing a much larger exodus than what is reported. Emigrants have a greater incentive to report their arrivals to the administration of the destination country (which is a prerequisite for accessing rights and social benefits, such as health services and education) than to the administration of their country of origin [23]. Furthermore, these public administrations do not share information with one another.

**Table 2:** Flow of Spanish migrants between 15 and 34 years old to foreign countries.

Country	2008	Country	2016
United Kingdom	2,044	United Kingdom	4,798
France	1,060	Germany	3,009
Germany	823	France	2,124
Switzerland	366	Switzerland	1,209
Ireland	362	Netherlands	490
Italy	338	Belgium	438
Netherlands	334	Ireland	394
Belgium	239	Italy	341
Portugal	159	Sweden	275
Denmark	85	Denmark	203
Norway	85	Norway	181
Sweden	60	Portugal	165

Data extracted from the Spanish National Statistics Institute (Instituto Nacional de Estadística INE) [6]

Since the beginning of the crisis, the mass media have reported on Spain’s complex economic, social and political circumstances. ‘Recession’, ‘unemployment’, ‘deficit’ and ‘brain drain’ are among the recurrent terms and expressions used in the morning news; their unquestioned presence has blurred much of the information received by Spanish people. Thus, a socialization strategy with a scope as broad as the entire population of Spain has been created by the mass media [24]. Because these phenomena have become common in our daily lives, the objective of this research is to analyze the media coverage of a particular phenomenon, namely, the Spanish ‘brain drain’, or ‘talent drain’, which refers to the emigration of young Spanish people to foreign countries to seek a better future.

To summarize, the introduction describes the importance, causes and social construction of the crisis; presents the global consequences, especially regarding the collective of young people; and highlights newly emerging phenomena, including ‘truncated careers’ [19] and the ‘brain drain’. In this context, the introduction highlights a need to develop systematic descriptors to quantify the characteristics of the press reports (such as *newspapers*, *years*, *sections* or specific *assessments*) regarding the emigration of young Spanish human capital from the beginning of the great recession to the present day. Revealing how this phenomenon is approached by the media by taking articles as the object of study entails highlighting the cultural and ideological qualities of the media [25] and remarking on their role in day-to-day socialization, keeping in mind that the media are not disinterested parties [25]. The proposed bibliometric approach [26, 27] allows us to identify patterns, changes and omissions in the large-scale coverage of the youth exodus, which is a necessary task for reflecting its circumstances and effects.

A bibliometric approach comprises a set of procedures and methods that quantify scientific literature for subsequent analysis [28]. In other words, bibliometrics is the use of statistical and mathematical techniques, such as text analysis and characteristic counting systems, to study the essence and development of written communication processes, scientific disciplines and thematic fields [29]. This methodology has been widely applied to quantify activities, structures and evolution in terms of productivity when analyzing authors, professional journals or academic disciplines, among other subjects [30].

## Method

The methodological layout of this research is inspired by the bibliometric guidelines designed by Selva, Sahagún and Pallarès [31]. The bibliometric approach used in this research entailed the codification of each article in terms of variables such as *section* and *assessment of the phenomenon*, among others, to allow their analysis through correspondence and frequency tables.

### Article selection

The corpus of the analysis is composed of a set of articles published in the press (both print and digital) about the emigration of young Spanish people and the labor market. The selection of the *newspapers* in which the search was conducted was based on the following criteria: a) periodicity, with daily publications prevailing; b) type of distribution, with digital publications with open access prevailing; c) average daily circulation, with higher-circulation publications prevailing; d) average daily spread, with widespread publications prevailing; e) average number of daily readers, with higher readership publications prevailing; and f) relevance and representativeness, with *newspapers* from the country of origin and the most frequent destination countries (or potential destination countries) prevailing.

The selection of the articles was based on two main criteria: a) the topic of the article was evidently the emigration of young Spanish people and the labor market and b) the article was published between 2007 and 2016, both years included (i.e., from the beginning of the Spanish economic and social crisis until the present day, with the latter defined as the last full calendar year).

The scanning, selection and storage processes applied to the corpus were performed in four phases. In the first phase, articles in the selected *newspapers* were searched via their web portals using advanced search engine tools that allowed us to a) define the temporal range (e.g., from 01/01/2007 to 12/31/2016); b) sort by thematic significance and/or date; and c) search for keyword combinations by using the Boolean operator ‘AND’ (e.g., young people AND emigration AND labor market AND professional expectations), which enabled us to narrow the search by linking terms and defining an additive relationship among them. In the second phase, the articles encountered were preselected when they matched the indicated thematic and temporal criteria. In the third phase, each full article was stored in Word format and named following the pattern ‘newspaper name’_‘assigned number’_‘publication year’ (e.g., Liberation_23_2010.doc), and its full reference was introduced into an Excel matrix. Finally, in the fourth phase, we performed a deep revision of the entire selected corpus with the goal of refining it by eliminating duplicates and articles not directly related to the topic. The corpus of 369 preselected articles was thereby reduced by 23 articles that were either duplicates and or did not directly relate to the topic, leaving 346 articles in the corpus.

### Codification

To define the set of variables for the study, a sample of 100 randomly selected articles from each newspaper was analyzed. To conduct this procedure, an initial codification was individually performed by a team of four researchers; once it was completed, each researcher presented for discussion a proposal regarding cases that could generate doubt or divergence. After a process of identification, discussion and systematization, it was developed a list that included four descriptive variables (*author or agency*, *publication year*, *newspaper* and *language*) and five categorical variables (*section*, *method*, *approach to the phenomenon*, *assessment of the phenomenon* and *overview of the phenomenon*), with each variable composed of various emerging analytical codes. It should be noted that whereas the descriptive variables remitted to the group of indicators that allowed us to identify the basic coordinates of each article (e.g., the *author* or the ideological orientation of the article based on the *newspaper* in which it was published), the categorical variables described prominent features, attributes or qualities of the article.

Throughout the identification and codification processes, the categorical variables required more attention because they needed a deeper examination of each article in terms of the treatment of the phenomenon and the focus of attention of the article. *Section* and *assessment of the phenomenon* were the most complex variables, as they also required thematic abstraction. For the *section* variable, we elaborated a preliminary list with all of the headings under which the selected newspapers commonly group their articles based on content; once adjusted to the selected corpus and thematically subsumed, the final list contained eight codes. The codification of the *assessment of the phenomenon* variable was slightly more complex because a preliminary list of themes or codes could not be elaborated; rather, themes and codes had to emerge from the examination of the articles and achieve a balance between specificity and amplitude. The result was an analytical framework configured by 14 codes. Due to the complexity of the category, the framework was restated to introduce an intermediate level of analysis, containing the subcategories *Loss of Capital*, *Personal Effect*, *Actions*, *Investment/Opportunity* and *Descriptive*, which enabled the data to be considered with a greater degree of precision because each article presented simultaneous aspects of *assessment of the phenomenon* (e.g., *Loss of Capital* and *Personal Effect*). With the aim of further refining the variable *assessment of the phenomenon* and attending to its specificity, an extra field ‘general comments’ was added to the Excel matrix, which allowed us to qualitatively address specific aspects that had driven the usage of a specific code and that later would help us to refine the definition by category and code.

In short, the resulting analytical framework (descriptive and categorical variables) comprises nine categories, five subcategories and twenty-nine codes.

### Analysis

Upon completion of the codification, a matrix containing all of the data was generated so it could be used in a statistical package (SPSS 24). The matrix was essentially processed using a descriptive approach, restricted to frequency tables (for the univariate analysis) and to contingency tables and correspondence analysis (for the bivariate analysis). The correspondence analysis was a fundamental element of the analysis of the relationships between *assessment of the phenomenon* and *section* and between *assessment of the phenomenon* and *year*.

## Results

The analytical corpus comprises 346 press articles published between 2007 and 2016 by nineteen newspapers: eight Spanish (268 articles) [32–39], five English (31 articles) [40–44], three French (29 articles) [45–47] and three German (18 articles) [48–50], which correspond to the country of origin (Spain) and the main destination countries (United Kingdom, France and Germany) of young Spanish emigrants.

As Table 3 shows, the number of articles rose moderately until 2010 and began to increase significantly in 2011, reaching maximum representation in 2013 with 25.72% and a cumulative percentage of 58.39%. The increase in the number of articles published regarding the studied phenomenon is notable because its singularity does not apply for recurrent articles, such as the several articles related to labor force surveys that are published every month. The lack of recurrent attention to topics related to the ‘truncated careers’ is mainly because the outcomes of this emigration are not fully evident immediately or in the short-term; rather, the repercussions (in the social, economic, educational and territorial fields) emerge in the medium or long term. For this reason, the number of articles has increased over the years as the consequences of emigration started to become palpable.

**Table 3:** Distribution of articles by year of publication.

*Publication Year*	Freq.	%	% Cumulative Freq.
2007	2	0.58	0.58
2008	4	1.16	1.74
2009	6	1.73	3.47
2010	7	2.02	5.49
2011	27	7.80	13.29
2012	67	19.36	32.65
2013	89	25.72	58.37
2014	52	15.03	73.40
2015	36	10.41	83.81
2016	56	16.19	100.00
Total	346	100.00	

If we divide the ten-year period covered by this study into two lustrums (see Table 4), we see that during the first five-year term (2007–2011), the Spanish press basically approached the phenomenon to show its effects, causes and repercussions from a regional and delimited point of view, as we would expect. However, in the second lustrum (2012–2016), the phenomenon becomes a recurring topic in the Spanish press, with a cumulative percentage of 85.98%, and a major topic in the international press, with a cumulative percentage of 89.34% (corresponding to 233 and 67 articles, respectively).

**Table 4:** Distribution of articles by year of publication and origin.

*Year*	Spanish Articles				International Articles			
	Articles	%			Articles	%		
2007	2	0.74			0	0.00		
2008	4	1.48			0	0.00		
2009	6	2.21			0	0.00		
2010	6	2.21			1	1.33		
2011	20	7.38	38	14.02	7	9.33	8	10.66
2012	43	15.87			24	32.00		
2013	67	24.72			22	29.34		
2014	40	14.76			12	16.00		
2015	31	11.44			5	6.67		
2016	52	19.19	233	85.98	4	5.33	67	89.34
	271				75			

To explore possible explanations for the increase in the number of published articles, we calculated Pearson’s correlation coefficient between the number of articles published and the emigration rate. Because the emigration rate to a specific destination country is not collected by either Eurostat or the INE (Spanish National Statistics Institute), it was calculated by dividing the number of Spanish migrants among the three countries with higher emigration (i.e., the United Kingdom, Germany and France) by the Spanish population on the first of January between 2008 and 2016. Both parameters were limited to Spanish people between 15 and 34 years old. All of these data and the number of articles published by year are shown in Table 5. Pearson’s correlation coefficient was calculated with the variables for emigration rate (per thousand) and number of articles published. The result, 0.5885, evinces a moderate positive correlation between them.

**Table 5:** Correlation between the emigration rate and number of articles.

Year	Population	Migrants	Emigration Rate	Articles
2008	10,590,554	3,927	0.37	4
2009	10,361,624	3,801	0.37	6
2010	10,100,618	4,242	0.42	7
2011	9,847,411	6,099	0.62	27
2012	9,580,489	6,101	0.64	67
2013	9,310,209	7,530	0.81	89
2014	9,095,563	9,101	1.00	52
2015	8,913,074	10,758	1.21	36
2016	8,734,518	9,931	1.14	56

Data extracted from INE [6] available only from 2008

Following that thread, we calculated Pearson’s correlation coefficient between the unemployment rate and the number of articles published. Table 6 shows the unemployment rates of Spanish people between 15 and 34 years old and the number of articles published by year. Pearson’s correlation coefficient for these two variables is 0.7915, evincing a positive strong linear correlation between them.

**Table 6:** Correlation between the unemployment rate and number of articles.

Year	Unemployment Rate	Articles
2007	8,23	2
2008	11,25	4
2009	17,86	6
2010	19,86	7
2011	21,39	27
2012	24,79	67
2013	26,10	89
2014	24,44	52
2015	22,06	36
2016	19,64	56

Data extracted from INE [6]

### Section

Although the *newspapers* selected for this study differ in terms of the specific *sections* into which they organize their content, these sections are more or less equivalent to one another (or differ only by name); thus, the thematic grouping resulted in eight categories: a) *society*, b) *economy*, c) *national*, d) *politics*, e) *international*, f) *opinion*, g) *education and jobs*, and h) *science and technology*. The thematic categories and their respective definitions are shown in Table 7.

**Table 7:** Content of each code under the section variable.

Code	Content
*Society*	Articles that cover social events and the social lives of certain individuals as well as other news that does not have a specific classification (e.g., religion, culture, etc.)
*Economy*	Articles that cover events from the commercial and business world (stock market, finance, marketing, companies, etc.)
*National*	Articles that cover the most relevant events in the local, regional or national scene (in this case, Spain)
*Politics*	Articles that present the most relevant news from the national and international political scenes
*International*	Articles that describe the most relevant events from the international scene (in this case, outside of Spain)
*Opinion*	Open-platform articles about current topics, including readers’ opinions (e.g., letters to the editor) and the opinions of the newspapers’ editors (editorial columns)
*Education and Job*	Articles related to the educational world (universities, schools, pedagogy, etc.) or work (companies, labor conflicts, office ergonomics, etc.)
*Science and Technology*	Articles related to the latest trends in technology, the Internet, social networks, scientific research, science, etc.

As can be seen in Table 8, the *society* and *economy sections* are those in which most of the articles regarding the studied themes were published, with these two *sections* accounting for 50.57% of the articles. Nevertheless, the high frequency of articles in all *sections* proves that the phenomenon prompted widespread investigations and was approached repeatedly from multiple points of view, with direct implications and repercussions in the social, political, economic, labor, technological, education, and vital service fields, among others.

**Table 8:** Distribution of articles by section.

*Section*	Freq.	%
*Society*	99	28.61
*Economy*	76	21.96
*National*	47	13.58
*Politics*	35	10.12
*International*	34	9.83
*Opinion*	23	6.65
*Education and Job*	18	5.20
*Science and Technology*	14	4.05
Total	346	100.00

### Method

For the *method* variable, the classification was made by identifying the logical processes through which the articles of the corpus collected their data. Three large typologies of data acquisition were identified: a) *Secondary Analysis of Data*; b) *Phenomenological Approach*; and c) *Survey*. The definitions of these typologies are shown in Table 9. Most articles in the corpus (58.09%) are based on *secondary analysis of data*, followed by articles using a *phenomenological approach* (33.24%) and those that use *survey* data (only 8.67%).

**Table 9:** Method categorical variables content.

Category	Content
*Secondary Analysis of Data*	Approached the phenomenon using data collected, structured and published by different public and private bodies (e.g., INE)
*Phenomenological Approach*	Used an interpretive approach to the phenomenon based on the subjective experience of the storyteller as told through interviews, opinions or thoughts
*Survey*	Examined public opinion regarding a phenomenon using a set of questions directed to a preselected target group

### Assessment of the phenomenon

As previously stated, the classification of the *assessment of the phenomenon* into thematic areas was inductive. In other words, the general principle of each area was defined based on the observations made during the pilot thematic classification. The categories, subcategories and thematic codes, as well as their respective definitions, are shown in Table 10.

**Table 10:** Categorical variables.

Category	Subcategory	Category definition	Codes	Code definitions
*Approach to the Phenomenon*	-	The perspective from which the emigration is analyzed	*Individual*	The phenomenon is analyzed or explained by focusing on a single person
			*Collective*	The phenomenon is analyzed or explained by focusing on a group of people
*Assessment of the Phenomenon*	*Loss of Capital*	Regarding the effects of Spanish migrants on a foreign country as a consequence of the cultural and socioeconomic crisis in Spain	*Intellectual*	Emigration of professional, scientific or higher educated people prompted by scarce opportunities for labor insertion or by the lack of development opportunities in their professional areas in their country of origin, also known as ‘brain drain’
			*Human*	Emigration of young people with productive capacity (older than 16 years) without formal education, with a medium level of education or without specified educational level
			*Economic*	Loss of educational investment and reduction of the number of future taxpayers and employees who can economically sustain the welfare state with their taxes and contributions
	*Personal Effect*	Truncated career consequences for a person	*Social Exclusion and Identity Crisis*	Impossibility of participating in the social, economic or cultural life of young people due to the lack of rights, resources and basic capacities as a result of being outside the labor market; includes difficulties in developing competences linked to labor experience that often are associated with serious doubts about oneself and the future
			*Labor Expectations and Failure*	Absence or change of personal expectations regarding the labor market, the chances of labor inclusion or opportunities for development
			*Future Perspective*	Confusion or absence of perspective regarding future life and chances of improvement
	*Actions*	Measures and initiatives with the aim of human and intellectual capital retention or comeback	*Retention*	Measures that have the objective of preventing the demographic drain of young people
			*Comeback*	Measures that have the objective of facilitating the comeback of the demographic drain of young people
			*Retention and Comeback*	Measures that have the objective both of preventing the demographic drain of young people and facilitating the comeback of the demographic drain
	*Investment/ Opportunity*	Emigration associated with the search for a curriculum development (education, language, expertise) or a challenging experience	*Labor Development Strategy*	Emigration with the purpose of easing the initiation or development of one’s professional career and motivated by the need to be self-sufficient or to develop a labor trajectory
			*Adventure*	Emigration oriented towards a life experience, usually with some aspect of uncertainty, seeking unexpected events and with a certain amount of risk
	*Descriptive*	Where the focus of attention on the emigration is; relevant and mentioned aspects	*Phenomenon Indicators*	Data, figures or statistics directly related to the phenomenon
			*Sociodemographic Indicators*	Data, figures or statistics directly related to the sector, segment or field
			*Sociodemographic and Phenomenon Indicators*	Data, figures or statistics directly related to the sector, segment or field and data, figures or statistics directly related to the phenomenon
*Overview of the Phenomenon*	-	Information, statistics or data about the phenomenon or a status report of the situation without assessment	*Causes*	Emphasis on the reasons behind the emigration: social, psychological (uncertainty, discomfort), economic, political, etc.
			*Consequences*	Emphasis on the effects or possible effects of the emigration on the country, person, generation, etc.

Note that in classifying the *assessment of the phenomenon* (and the *overview of the phenomenon*), each article could have been assigned more than one code (e.g., *social exclusion and identity crisis* and *sociodemographic indicators* for *assessment of the phenomenon* and *causes* and *consequences* for *overview of the phenomenon*).

Table 11 shows the distribution of articles in terms of the *assessment of the phenomenon*: 37.77% focused on *loss of capital*; 20.89% focused on *personal effect*; 20.31% tended to be more *descriptive* by mentioning *indicators*; 13.02% approached the phenomenon in terms of *investment/opportunity*; and the remaining 8.01% discussed *actions* or measures.

**Table 11:** Distribution of articles by assessment of the phenomenon.

*Assessment of the Phenomenon*	Freq.	%
***Loss of Capital***	**264**	**37.77**
*Intellectual* *Human* *Economic*	*(171)* *(64)* *(29)*	*(64*.*77)* *(24*.*24)* *(10*.*99)*
***Personal Effect***	**146**	**20.89**
*Labor Expectations and Failure* *Future Perspective* *Social Exclusion and Identity Crisis*	*(88)* *(40)* *(18)*	*(60*.*27)* *(27*.*40)* *(12*.*33)*
***Descriptive***	**142**	**20.31**
*Sociodemographic Indicators* *Phenomenon and Sociodemographic Indicators* *Phenomenon Indicators*	*(52)* *(49)* *(41)*	*(36*.*62)* *(34*.*51)* *(28*.*87)*
***Investment/Opportunity***	**91**	**13.02**
*Labor Development Strategy* *Adventure*	*(87)* *(4)*	*(95*.*60)* *(4*.*40)*
***Actions***	**56**	**8.01**
*Comeback* *Retention* *Retention and Comeback*	*(25)* *(19)* *(12)*	*(44*.*64)* *(33*.*93)* *(21*.*43)*
**Total**	**699**	**100.00**

More specifically, most articles that assess the phenomenon in terms of *loss of capital* do so by mentioning *intellectual* capital (64.77%), which is also referred to as ‘brain drain’, followed by those that focus on the loss of *human* capital (24.24%) and those that highlight the *economic loss of capital* produced by the phenomenon (10.99%). Regarding the impact or consequences of the phenomenon that relate to *personal effects*, the majority of articles (60.27%) describe the absence of *labor expectations*, which provokes a feeling of *failure* in those affected by it; smaller number of articles highlight fear and concern about the *future perspective* and other harsh consequences such as *social exclusion and identity crisis* (27.40% and 12.33%, respectively). Numerous articles tackle the phenomenon at a *descriptive* level, focusing their interest on *indicators* that measure or highlight changes; among them, 36.62% focus on *sociodemographic indicators*, 34.51% on both *phenomenon* and *sociodemographic indicators*, and the remaining 28.87% only on *phenomenon indicators*. Regarding articles that analyze the emigration in terms of *investment* or *opportunity*, nearly all of them (95.60%) associate it with a *labor development strategy* as a thoughtful course of action undertaken in search of professional improvement; in contrast, only 4.40% describe a desire to experience an *adventure* outside one’s comfortable daily life. The last group of articles relate *actions* and measures taken or considered by public organizations to help the emigrants to *comeback* (44.64%), to prevent others from emigrating by promoting *retention* motivations (33.93%), or to accomplish a combination of *retention* and *comeback actions* (21.43%) in an effort to recover the lost social structure.

**Fig 1:**
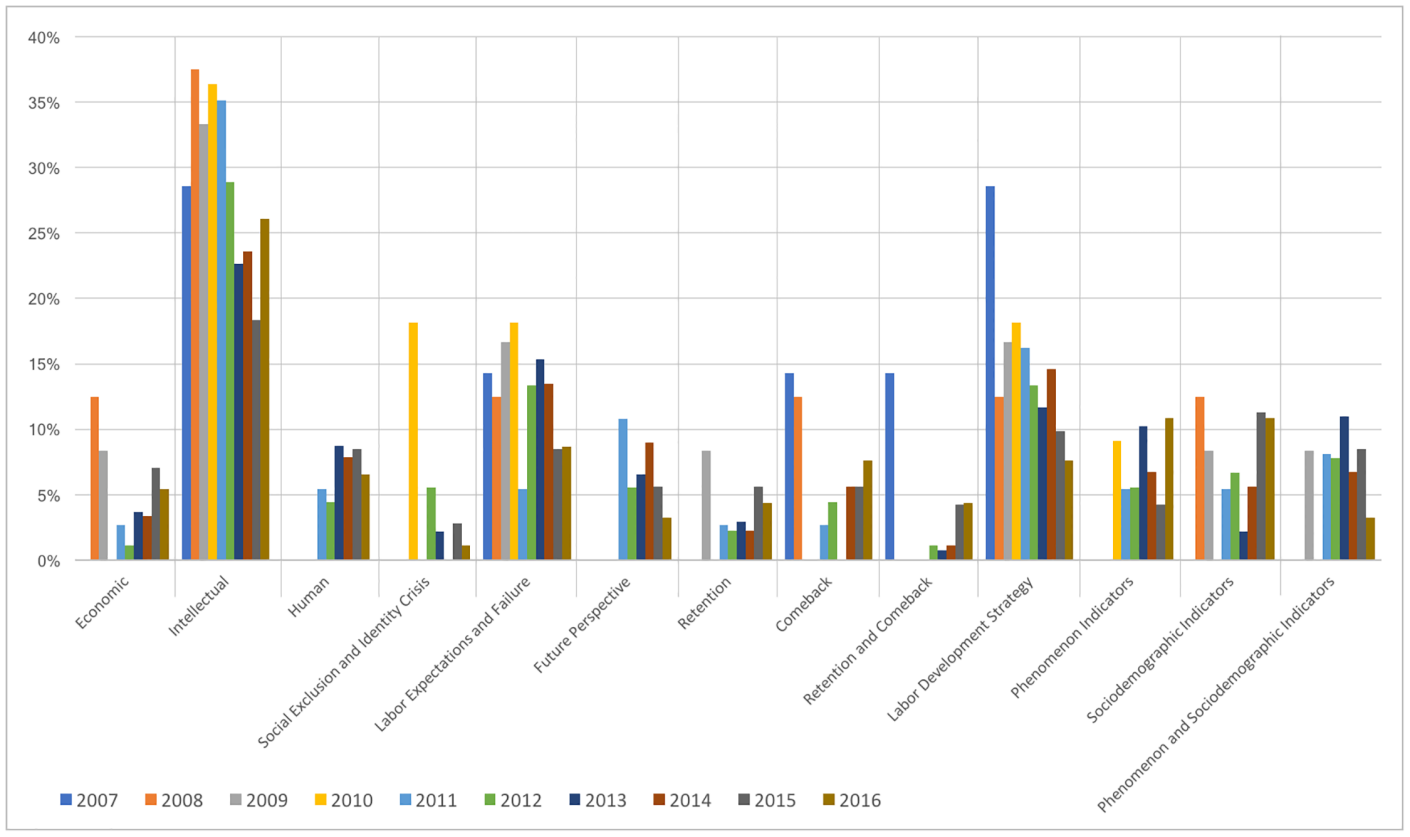
Spanish assessment of the phenomenon proportion by year.

From another perspective, Fig 1 shows, for Spanish newspapers, the weight of each code in relation to the total. This weight is calculated as the number of times each code appeared divided by the total number of times the code was mentioned each *year* and is represented graphically in the figure. This figure illustrates the evolution of the presence of each code under the categorical variable *assessment of the phenomenon* in the newspapers by *year*. Fig 1 is configured by thirteen of the fourteen codes (*adventure* is not displayed due to its scarce presence) and shows the percentage of articles regarding each code in comparison to the total articles for the *year*, grouping all of the data for each code differentiated by *year*. Thus, we can clearly see that loss of *intellectual* capital is, without a doubt, the code with the highest presence during the ten-year period of study; *labor expectations and failure* and *labor development strategy* also stand out. In other words, during the crisis, the central focus of articles of Spanish newspapers related to the emigration was the loss of talented young people (also called ‘brain drain’), which is linked to the absence of labor expectations (or, alternatively, a feeling of failure) and to the implementation of development strategies (in this case, emigration) as a way to start or develop a professional career. It is also apparent that over the years, the focus of the news shifted to the *loss of capital* as the loss of productive capacity of the lost collective, without considering its qualifications, for the Spanish labor market; the coverage included data, figures and statistics that illustrated the loss.

By conducting an in-depth examination of the weight and evolution of each code in relation to the others, we can see in Fig 1 that *economic*, *comeback* and *retention and comeback* behave similarly. Specifically, they appear at the beginning of the crisis, accounting for over 10% of total coverage; then, their presence is reduced to a negligible level or even disappears; finally, in the last two years, they achieve some level of presence. *Retention* exhibits a similar behavior, except that it first appears in the third year of the crisis. The code *Intellectual* has the largest presence, but its weight changes significantly over the passage of time; in the first five years following the beginning of the crisis, it accounts for approximately 35% of the total, whereas in the second lustrum, it decreases by more than 10 percentage points. This change is further evidence that at the beginning, the primary focus was on talented and/or more educated emigrants, but over the years, the crisis expanded to include the entire young collective. The *human*, *future perspective* and *phenomenon indicators* codes do not appear in the first 3–4 years of the crisis; in the remaining years, they have a sustained presence between 5% and 10%. *Labor expectations and failure* and *labor development strategy*, as two of the relevant codes, behave similarly, with a continued presence of over 10% during the period of study and a slight reduction in the last two years (2015–2016), probably because the first is one of the obvious and more common causes of the phenomenon and the second is the main consequence of it. Some of the weight over the last two years is taken by *sociodemographic indicators*, which has a substantial presence at the beginning of the phenomenon and in the last two years of the study. This code is present because these *indicators* offer rapid information when a tendency clearly changes. Consider, for example, the unemployment rate: its level and trend are stable, then a sustained change in it becomes a clear indication of a new situation. Therefore, it is reasonable that the press would highlight such indicators. The code that combines *phenomenon and sociodemographic indicators* has a stable weight of over 5%, with sporadic years of non-appearance because certain articles focus only on *phenomenon indicators* or on *sociodemographic indicators* without linking the two. Finally, the *social exclusion and identity crisis* code exhibits uneven behavior because of its specificity.

**Fig 2:**
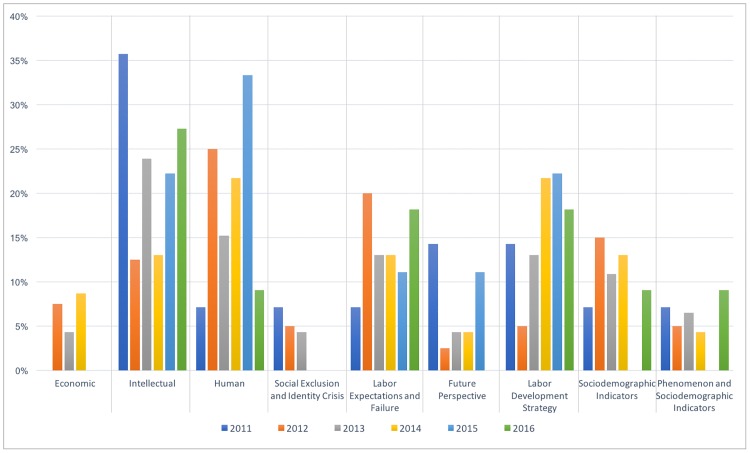
International assessment of the phenomenon proportion by year.

To create Fig 2, we followed the same procedure as for Fig 1 but using articles published in international newspapers in order to show each code’s presence during the evolution of the categorical variable *assessment of the phenomenon*. Fig 2 is configured by nine of the fourteen codes (*adventure*, *phenomenon indicators*, and the *actions* codes *comeback*, *retention*, *and retention and comeback* were excluded because they are only rarely mentioned). The lower number of codes in Fig 2 is to be expected because certain codes are closely related to Spain and the phenomenon; for example, the fact that Spanish public organizations are initiating measures to retain or help emigrants to return is not a relevant theme for international newspapers. Accordingly, we can see that there are no articles between 2007 and 2010, which is understandable given that the phenomenon originated in Spain and did not become newsworthy outside of Spain until several years later.

Fig 2 shows that *intellectual* and *human loss* of *capital* have similar levels of presence, in contrast to the Spanish newspapers, but are not significantly more present than *labor development strategy* and *labor expectations and identity crisis*, which are also prominent. In other words, international newspapers did not focus exclusively on the aforementioned ‘brain drain’ but approached the phenomenon from a broader perspective, giving similar weights to the characteristics of the emigration (*human* and *intellectual*) and to the reasons for it (absence of *labor expectations* and *labor development strategy*). It is difficult to extract more information from international newspaper because, as shown in Table 4, more than 77% of the articles in international newspapers appeared between 2012 and 2014, which suggests that the phenomenon is not considered critical in the destination countries.

### Approach to the phenomenon and overview

The last two categorical variables are *approach to the phenomenon* and *overview of the phenomenon*. The frequency of the first variable is an evidence that the phenomenon has mainly been treated as *collective*, with 76.09%, compared with reports focused on the *individual*, which account for 23.91%. This finding implies that the phenomenon has been addressed by considering information for a large number of people and not by focusing on the opinions or feelings of *individuals*; the focus on the *collective* reinforces the phenomenon and decreases the likelihood that the coverage is skewed because it is not based on subjective views. The second variable indicates that both the *causes* (43.23%) and *consequences* (57.77%) of the phenomenon have been highlighted; the frequent mention of both its origins and its effects is a positive sign because it shows awareness of the reasons for the phenomenon and recognition of the fact that its effects will be present for a long time and that it has affected (or will affect) many areas.

### Correspondence analysis

The dependency relationships between categorical, or nominal, variables were studied using a correspondence analysis. This multivariate technique, in its multiple correspondence mode, allows us to examine the quality and magnitude of the associations between the objects in the rows (press articles) and the objects in the columns (variables) by converting the contingency tables to maps with the consequent statistics calculations [28, 51]. In particular, the correspondence distribution was used to examine the relationships between *assessment of the phenomenon* and *year* and between *assessment of the phenomenon* and *section*.

#### Assessment of the phenomenon and year

**Fig 3:**
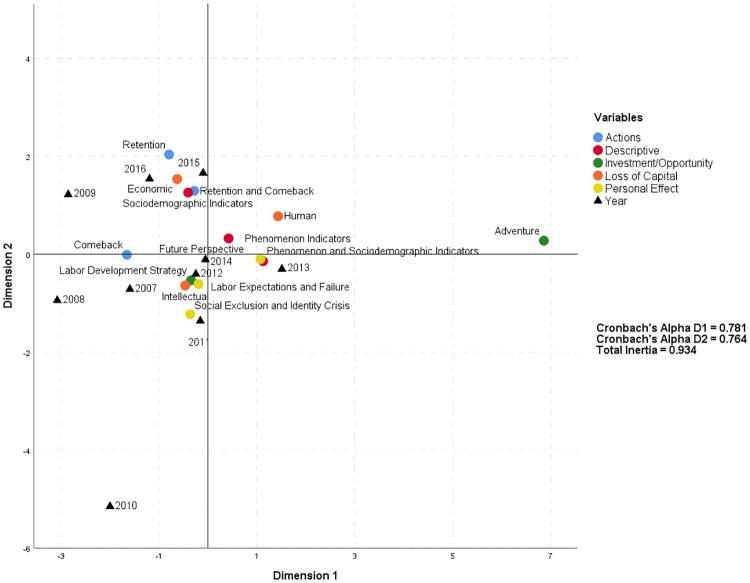
Correspondence analysis of assessment of the phenomenon and year.

Table 12 shows the dimensions and coordinates of the analysis from which the Cartesian diagram (Fig 3) showing the association between the variables *assessment of the phenomenon* and *year* is derived.

**Table 12:** Correspondence coordinates between assessment of the phenomenon and year.

***Year***	Frequency	Dimension	
		1	2
2007	2	-1.593	-0.714
2008	4	-3.077	-0.944
2009	6	-2.854	1.215
2010	7	-1.999	-5.147
2011	27	-0.156	-1.360
2012	67	-0.248	-0.407
2013	89	1.509	-0.305
2014	52	-0.052	-0.113
2015	38	-0.098	1.656
2016	54	-1.193	1.536
***Loss of Capital***	Frequency	1	2
*Economic*	29	-0.629	1.532
*Intellectual*	171	-0.465	-0.639
*Human*	64	1.432	0.774
***Personal Effect***	Frequency	1	2
*Social Exclusion and Identity Crisis*	18	-0.361	-1.226
*Labor Expectations and Failure*	88	-0.194	-0.609
*Future Perspective*	40	1.071	-0.099
***Actions***	Frequency	1	2
*Retention*	19	-0.794	2.033
*Comeback*	24	-1.654	-0.012
*Retention and Comeback*	12	-0.281	1.292
***Investment/Opportunity***	Frequency	1	2
*Labor Development Strategy*	87	-0.344	-0.534
*Adventure*	4	6.859	0.276
***Descriptive***	Frequency	1	2
*Phenomenon Indicators*	41	0.421	0.321
*Sociodemographic Indicators*	52	-0.404	1.256
*Phenomenon and Sociodemographic Indicators*	49	1.126	-0.143
**Model summary**			
Dimension	Cronbach’s alpha	Self-value	Inertia
1	0.781	2.864	0.477
2	0.762	2.739	0.456
Total		5.603	0.934
Mean	0.772^a^	2.801	0.467

^a^. The mean of Cronbach’s alpha is based on its self-value mean

Fig 3 shows a points cloud in which the proximity of several relationships between *years* and certain *assessments of the phenomenon* are notable. In hindsight, it is apparent that in the last *year* of the study, 2016, the following themes were of greater interest to the written media: *retention* and *comeback actions* for young people, the impact of the loss of *economic* capital and *sociodemographic indicators*. As could be expected, a decade after the beginning of the crisis and following the emigration wave of young capital (which reached its highest level in 2015 with 36,238 registered departures; see Table 1), the focus has shifted to the consequences of the migration exodus and to *actions* that seek the *retention* and *comeback* of emigrants. The preceding *year*, 2015, shows a similar trend, with the addition of the loss of the *human* capital and productive capacity of the emigrated collective. In 2014, which situated very close to the coordinates’ origin, and 2013, which is to the right of 2014 in the inferior quadrant, are related to *descriptive* themes regarding young people’s situations based on *phenomenon* and *sociodemographic indicators* and on the individual impact of the *future perspective*. In other words, in these years, sociodemographic and macroeconomic signs related to the phenomenon became apparent. For example, the tripling of the unemployment rate and its impact on the personal situations of young people was reflected in insecurity and uncertainty about the future. In 2012, frequent topics were the loss of *intellectual* capital, *labor expectations and failure* and *labor development strategies*. Particularly, after the increase of poverty risk and social exclusion rates started in 2008, young people, especially the most talented among them, developed strategies to begin or evolve their professional trajectories, which often entailed emigration from Spain because of the absence of labor opportunities there. The codes of 2012, adding *social exclusion and identity crisis*, are also associated with 2011 and 2010. Notably, 2010 is clearly located far from the main points cloud due to its scarce corpus; however, when we examine its universe of articles, we find the *assessments of the phenomenon* mentioned therein. From these associations, which are analogous to those mentioned in the previous period, emerge the first collateral effects of the crisis on the more highly trained young collective; i.e., they had little participation in the social, economic and cultural life of the country because of the shortage of rights, resources and basic capacities due to their exclusion from the labor market. Finally, the triad of 2009, 2008 and 2007, the incipient years of the crisis, are associated in a reiterative manner with the loss of *intellectual* capital, linking the emigration almost exclusively to highly skilled or talented young people, or the aforementioned ‘brain drain’.

In sum, Fig 3 provides a glimpse of the trends in the assessment of the phenomenon: a) the area of interest changes over the years, shifting from the young talented collective to the general collective, and b) this change is associated with the holistic treatment that the phenomenon received, moving from its characterization, identity conceptualization and effects to the purposeful concept of *actions* with a resolution to slow and reverse the situation.

#### Assessment of the phenomenon and section

**Fig 4:**
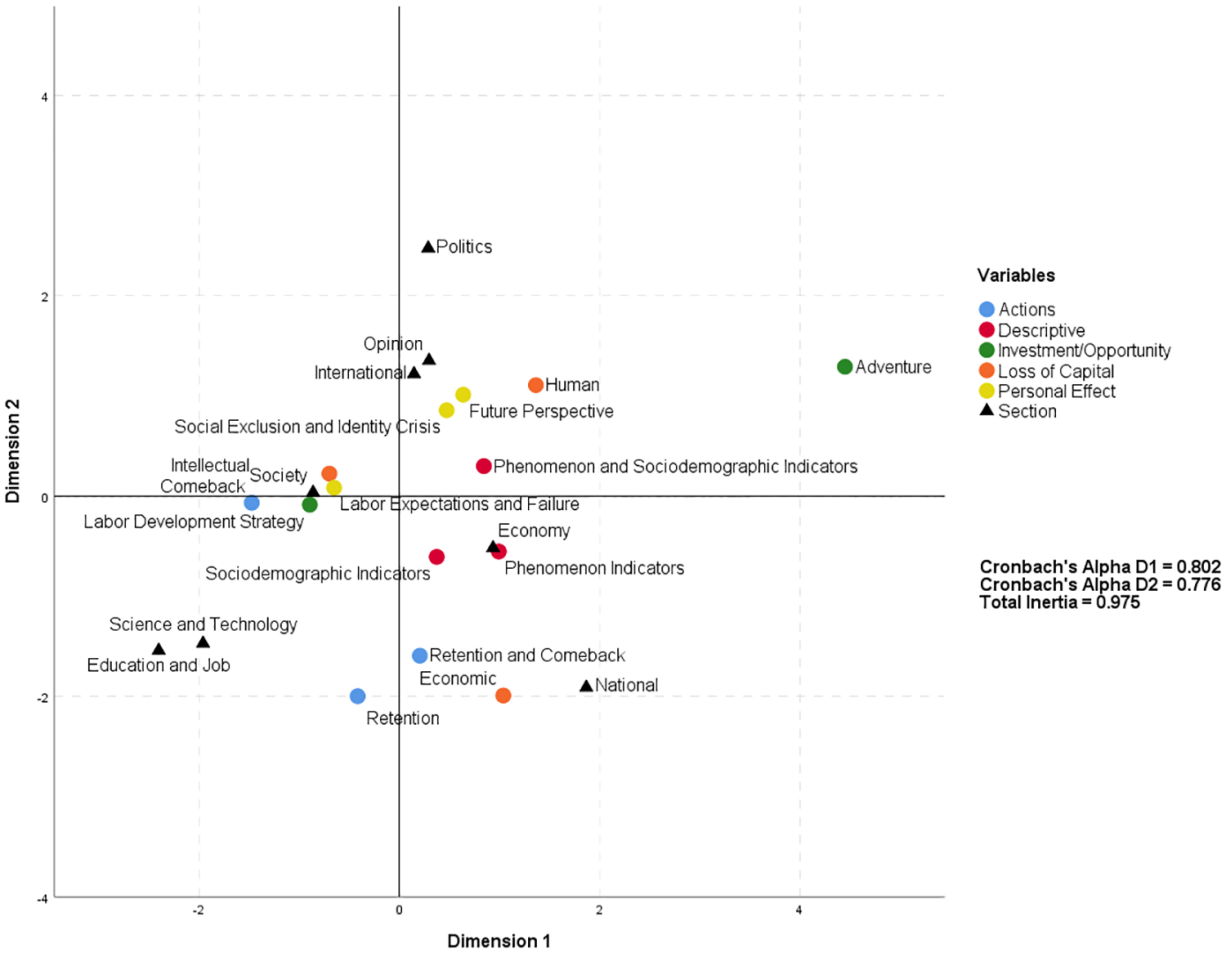
Correspondence analysis of assessment of the phenomenon and section.

Table 13 shows the dimensions and coordinates of the analysis from which the Cartesian diagram (Fig 4) showing the association between the variables *assessment of the phenomenon* and *section* is derived.

**Table 13:** Correspondence coordinates between assessment of the phenomenon and section.

***Section***	Frequency	Dimension	
		1	2
*Economy*	76	0.935	-0.518
*National*	47	1.865	-1.910
*Society*	99	-0.860	0.036
*Opinion*	23	0.296	1.353
*Education and Job*	18	-2.402	-1.540
*International*	34	0.146	1.220
*Science and Technology*	14	-1.959	-1.470
*Politics*	35	0.288	2.475
***Loss of Capital***	Frequency	1	2
*Economic*	29	1.038	-1.990
*Intellectual*	171	-0.699	0.225
*Human*	64	1.362	1.109
***Personal Effect***	Frequency	1	2
*Social Exclusion and Identity Crisis*	18	0.472	0.858
*Labor Expectations and Failure*	88	-0.651	0.084
*Future Perspective*	40	0.638	1.013
***Actions***	Frequency	1	2
*Retention*	19	-0.416	-1.997
*Comeback*	24	-1.473	-0.067
*Retention and Comeback*	12	0.205	-1.593
***Investment/Opportunity***	Frequency	1	2
*Labor Development Strategy*	87	-0.893	-0.085
*Adventure*	4	4.448	1.292
***Descriptive***	Frequency	1	2
*Phenomenon Indicators*	41	0.992	-0.553
*Sociodemographic Indicators*	52	0.373	-0.606
*Phenomenon and Sociodemographic Indicators*	49	0.843	0.300
**Model summary**			
Dimension	Cronbach’s alpha	Self-value	Inertia
1	0.802	3.019	0.503
2	0.776	2.829	0.471
Total		5.847	0.975
Mean	0.790^a^	2.924	0.487

^a^. The mean of Cronbach’s alpha is based on its self-value mean

Fig 4 shows a points cloud in which the polarity between *section* and *assessment of the phenomenon* is evident. The first association group is located in the top left quadrant, close to the horizontal axis, where are located the loss of *intellectual* capital, *labor expectations and failure*, *labor development strategy* and *comeback actions*, which are gathered in the *section society* because all of these themes have a strong social factor and great significance in daily life. The themes loss of *human* capital, *future perspective*, *social exclusion and identity crisis* and *phenomenon and sociodemographic indicators* are located in the top right quadrant near the *opinion* and *international sections*, which basically offer a snapshot of the general characteristics of the phenomenon (the affected collective and the main macroeconomic and sociodemographic signs that explain it). The *education and job* and *science and technology sections* are located in the bottom left quadrant and are related to the codes *retention* and *retention and comeback*. As could be expected, these *sections* generally lean toward the professional and work fields, tending to include articles that describe initiatives or plans to prevent the demographic drain of young people or to facilitate their comeback. Finally, the bottom right quadrant presents the main codes related to the *economy section*, including *phenomenon indicators* and *sociodemographic indicators* and the main code related to the *national section*, namely, *economic* capital. These relationships expose the tendency of the commercial and business *sections* to highlight the macroeconomic and sociodemographic aspects of the phenomenon. Nevertheless, the *sections* relating to local, regional and national spheres are focused on the consequences of the migration exodus, particularly the lost investment in education and the reduction in the number of future taxpayers and employees who can economically sustain the welfare state through their taxes and contributions.

In sum, Fig 4 provides a glimpse of the broad range of interests and impacts associated with the phenomenon in various *sections*, which based on their objectives highlight different *assessments*. In general, we can say that a) the phenomenon has had a significant impact on daily life and public opinion; b) the *actions* proposed to stop the exodus or stimulate the *comeback* basically originate in the fields of education and employment and professionalization; c) the effects on the economy related to the investment in education and its corresponding return on investment (ROI) are perceptible on a national scale and are substantial at the autonomous community level; and d) the phenomenon has had a substantial impact on the rates and indexes referred to by international media.

## Discussion

The crisis and the austerity policies promoted in recent years by the Spanish government have contributed to the emigration of young Spanish people, which has become a newsworthy phenomenon for the general mass media, particularly the written press. In these media, news, interviews, opinion articles and other journalistic productions regarding the social, economic, labor, collective and day-to-day situations of young people have proliferated, reaching their highest coverage share in 2013. This coverage suggests that despite widespread speeches delivered primarily by members of the Spanish government about economic recovery and the (progressive) recovery from the recent recession [52], the phenomenon and its aftermath continue to devastate Spanish society. One needs only to look at the emigration numbers to see that the phenomenon has persisted: the official census of 2016 shows that 9,531 people between 15 and 34 years old left Spain for one of the three countries that are the main recipients of Spanish capital. The statistical blur surrounding the phenomenon and the frequent partial and/or inaccurate interpretations of the data published by official sources has led to a clear undervaluation of these figures [23].

Facing a horizon of scarce opportunities—or opportunities accompanied by questionable labor conditions [8] that are more akin to unemployment than to being employed [13]—a large number of young people have chosen to leave the country as a subversive way to solve the problem of ‘truncated careers’ [19]. According to other studies [23], this migratory movement differs from former Spanish emigration waves because of the high female presence among the emigrants and their high levels of education, although the main destinations are the same: United Kingdom, Germany and France have always been typical European destinations for emigrants. The tendency to emigrate in search of (new) opportunities is a proof of the new configuration of labor markets and productive sectors and of the macroeconomic rebalancing [4]—whereby countries such as Spain, who are severely affected by the crisis and experiencing critical financial situations—effectively drive out their labor force while other countries, whose economic and productive systems have suffered less harm, benefit from the structural instability by attracting this workforce and strengthening their economic and political competitiveness [3].

The results of the descriptive analysis offer some interesting points to consider: a) there is recurrent publication in the press of articles regarding the phenomenon rather than the more gradual and discrete publication of scientific and specialized literature (the immediacy of the first is due to the need of the mass media to ‘live the news’, whereas scholarly literature is characterized by a slower pace and standardized channels and processes of acceptance and publication); b) the number of articles is strongly correlated with the unemployment rate, indicating that an increase (decrease) in the unemployment rate produces a corresponding increase (decrease) in the number of articles published on the topic; c) a frequent concern about the loss of *intellectual* capital, or ‘brain drain’ [12], terms that are sometimes improperly or randomly used to refer to any young people with productive capacity (*human* capital) rather than to young people who are highly educated and talented (its proper application); and d) there is a tendency to address the phenomenon using a *collective* approach (which avoids the possibility of bias that might be derived from personal stories but does not avoid biases derived from incorrect or opaque interpretations of data) and to consider both its *causes* and its *consequences*.

Although most of the articles appear in the *society* and *economy sections*, the high frequency of reports in other *sections* indicates that the phenomenon has multiple implications, which inevitably leads to talk of a holistic crisis (i.e., not only economic) or of a simultaneity of crises [3]. Most articles approach the phenomenon using published data (Instituto Nacional de Estadística [National Institute of Statistics], Eurostat, inter alia) in an effort to endow the subject with a certain halo of objectivity, but there is a growing trend toward the phenomenological approach (particularly during the periods in which the Spanish labor market is at its highest levels of precariousness), which brings us closer to the feelings and experiences of the story’s main characters.

The written press shows a heterogeneous interest in different themes or *assessments of the phenomenon*. The most common (and thus the most often published) themes are those related to the *loss of capital* and *personal effects*, which account for almost 60% of this category, followed by certain descriptive variables, strategies as *investments or opportunities* and *retention and comeback actions*. All these *assessments* enhance the new milestones and emerging phenomena of this collective, such as ‘youth prolongation’ [14], the construction of new identities and stories [15] about ‘being young’ [16], and the previously mentioned ‘truncated careers’ [19].

The analysis of the correspondence between *assessment of the phenomenon* and *year* shows that the focus has shifted from the singularity (with the loss of *intellectual* capital as the most relevant phenomenon in the first years of the study), macroeconomic constraints, and labor conditions to the collectivity (with loss of *human* capital gaining relevancy as time passes), consequences [20] and subversive methods. In the final period, which coincides with the time span in which a wide range of evidence emerged regarding the costs of the ‘youth exodus’ [21], *retention* and *comeback* measures started to receive increased attention from the media; however, their presence in the press is focused more on complaints regarding their absence than on their existence or success.

The correspondence between *assessment of the phenomenon* and *section*, in addition to providing insight into the validity and suggestive value of the phenomenon in the different *sections*, denotes the need to develop subversive proposals to stop the exodus and stimulate emigrants’ comeback, which is a relevant subject in the education, labor and professional *sections* (e.g., as a way to stop the waste of Spanish education). However: a) the reality experienced in Spain over the past decade, which has been particularly devastating to the young population due to precarious structural conditions [7]; b) the mid-term and long-term repercussions of the phenomenon that are already evident [20]; and c) the mass media pressure for action measures that scarcely exist; are increasingly pushing the Spanish government, particularly public and autonomous community institutions, to deploy *actions* and measures to stop the emigration wave, stimulate the emigrants’ comeback, and reduce the economic impact of the emigration phenomenon (good examples are the ‘QdaT’ or ‘Stay’ program launched by the Technical University of Valencia; European initiatives such as the Youth Guarantee Plan; *comeback* campaigns such as ‘Emigrated youth: Looking back home’ from the Spanish Youth Council; and websites that facilitate emigrants’ *comeback*, such as volvemos.org.).

## Conclusions

Beyond the specific *assessments*, *sections*, *approaches* and *methods* with which the phenomenon is approached, the media coverage of the emigration of Spanish youth highlights the widespread social concern about the phenomenon. Taking newspapers as an object of study enhances awareness of the attributes of the written press in terms of its function as a generator of day-to-day opinions and its role in socialization related to the phenomenon (which may be as diverse and uneven as the number of *newspapers* scrutinized). Considering the broad media coverage of the youth exodus, the numerous aspects of the phenomenon covered by the written press, and the cultural, ideological and social attributes of the emigration, the authors of this study believe that it is necessary to go a step further and analyze the specific language used in the selected articles. A critical analysis of the content—that is, what is said regarding the emigration of young Spanish people looking for a brighter future and how it is said—would challenge current discourses (particularly those delivered by the Spanish government to appeal to an adventurous spirit or the impulses of young people to emigrate) with the words of emigrants, describe the social constructions of the phenomenon, and note the mainstream qualities of the written press as communication media.

Furthermore, it is necessary to focus in depth on the ‘truncated careers’ of young Spanish people using a phenomenological approach that gives voice to the main characters and permits us to understand their trajectories, the discrepancies between their expectations and reality, and the individual strategies undertaken (such as emigration or resilience) to subvert or adapt to the situation.

## Supporting information

S1 File Supporting information. (XLSX)
